# Ambulance Service Utilization by Kidney Transplant Recipients

**DOI:** 10.1177/20543581251324587

**Published:** 2025-04-04

**Authors:** Kaveh Masoumi-Ravandi, Amanda Vinson, Aran Thanamayooran, Judah Goldstein, Thomas Skinner, Karthik Tennankore

**Affiliations:** 1 Division of Nephrology, Dalhousie University, Halifax, NS, Canada; 2 Division of Emergency Medical Services, Dalhousie University, Halifax, NS, Canada; 3 Department of Urology, Dalhousie University, Halifax, NS, Canada

**Keywords:** transplants, ambulances, emergency service, hospital, kidney

## Abstract

**Background:**

Compared with the general population, kidney transplant recipients (KTRs) frequently visit the emergency department (ED), but much less is known about the characteristics of ED presentations requiring ambulance transport and the impact on subsequent outcomes for KTRs.

**Objectives:**

To identify predictors of ambulance transport to the ED (ambulance-ED) and outcomes (graft failure and mortality) for those who experienced an ambulance-ED event in a cohort of KTRs.

**Design:**

Retrospective cohort study of incident, adult KTRs receiving a transplant from 2008 to 2020.

**Setting:**

Nova Scotia, Canada.

**Patients:**

Adult (≥18 years), Nova Scotian KTRs affiliated with the Atlantic Canada Multi-Organ Transplant Program.

**Measurements:**

Ambulance-ED events were captured for all transplant recipients (following the day of discharge from their initial transplant admission) using electronic records (provided by Emergency Health Services, the sole provider of emergency medical services for Nova Scotia). Ambulance-ED was defined as ambulance transport to the ED following a 911 call; interfacility transfers were excluded. Predictors of ambulance-ED included recipient, donor, immunological, and perioperative characteristics (pertaining to the initial admission for kidney transplantation). Outcomes included graft failure and mortality.

**Methods:**

Predictors of ambulance-ED were analyzed using a multivariable negative binomial regression model and reported using incidence rate ratios (IRRs) and 95% confidence intervals (CIs). The risk of death/graft failure for those with an ambulance-ED within 30 days of hospital discharge following transplantation was analyzed using an adjusted Cox survival analysis and reported using hazard ratios (HRs) and 95% CIs.

**Results:**

A total of 418 patients received a transplant during the study period. A total of 179 (42.8%) experienced one or more ambulance-ED events. Female sex (IRR = 1.60; 95% CI = 1.12-2.29), kidney failure secondary to diabetes (IRR = 2.52; 95% CI = 1.19-5.31), and donor age ≥45 (IRR = 1.50; 95% CI = 1.04-2.15) were all associated with ambulance-ED. There was no significant increase in the risk of death/graft failure for those that experienced ambulance-ED within 30 days of hospital discharge following transplantation (HR = 1.31; 95% CI = 0.44-3.94).

**Limitations:**

A limitation of this study was that ambulance-ED is not a perfect surrogate marker of acute care needs in a population. Important determinants of health such as living situation and socioeconomic status were not available in this data set.

**Conclusions:**

This study highlights the burden of ambulance use for KTRs and provides insight into the need for more optimal follow-up in certain patient subgroups who are at particularly high risk.

## Abrégé

**Contexte:**

Les receveurs d’une transplantation rénale se rendent plus fréquemment aux urgences (URG) que la population générale. On en sait toutefois peu sur ce qui caractérise les visites aux urgences avec transport en ambulance et sur les répercussions de ces événements sur les résultats des receveurs d’une transplantation rénale.

**Objectifs:**

Identifier les facteurs prédictifs d’une visite aux urgences avec transport par ambulance (événement ambulance-URG) et examiner les résultats (défaillance du greffon, décès) de ces événements dans une cohorte de receveurs d’une greffe rénale.

**Type d’étude:**

Étude de cohorte rétrospective portant sur des patients adultes ayant reçu une greffe de rein entre 2008 et 2020.

**Cadre:**

Nouvelle-Écosse, Canada

**Sujets:**

Des adultes (≥18 ans) transplantés du rein, affiliés au Atlantic Canada Multi-Organ Transplant Program, en Nouvelle-Écosse.

**Mesures:**

Les événements ambulance-URG de tous les receveurs d’une greffe rénale (à partir de la date du congé de leur admission pour la greffe) ont été répertoriés dans les dossiers électroniques (fournis par les Services d’urgence de santé, le seul fournisseur de services médicaux d’urgence en Nouvelle-Écosse). Un événement ambulance-URG a été défini comme un transport aux urgences par ambulance à la suite d’un appel au 911; les transferts entre établissements ont été exclus. Les prédicteurs d’un événement ambulance-URG comprenaient les caractéristiques des receveurs et des donneurs, ainsi que les caractéristiques immunologiques et périopératoires (relatives à l’admission initiale pour la transplantation). Les résultats comprenaient la défaillance du greffon et le décès.

**Méthodologie:**

Les prédicteurs d’un événement ambulance-URG ont été analysés à l’aide d’un modèle de régression binomiale négative multivariée, et présentés sous forme de rapports des taux d’incidence (RTI) avec intervalles de confiance à 95 % (IC95). Le risque de décès ou de défaillance du greffon des personnes ayant eu un événement ambulance-URG dans les 30 jours suivant la sortie de l’hôpital après la transplantation a fait l’objet d’une analyse de survie de Cox ajustée et rapporté sous forme de rapports de risque (RR) avec IC à 95 %.

**Résultats:**

Au cours de la période étudiée, 418 personnes ont reçu une greffe de rein et 179 (42 %) d’entre elles ont vécu au moins un événement ambulance-URG. Les caractéristiques qui ont été associées à un événement ambulance-URG sont: le sexe féminin (RTI: 1,60; IC95: 1,12–2,29), l’insuffisance rénale secondaire au diabète (RTI: 2,52; IC95: 1,19–5,31) et l’âge (≥45 ans) du donneur (RTI: 1,50 95 % 1,04–2,15). Aucune augmentation significative du risque de décès ou de défaillance du greffon n’a été observée chez les patients qui avaient subi un événement ambulance-URG dans les 30 jours suivant la sortie de l’hôpital après la greffe (RR: 1,31; IC95: 0,44-3,94).

**Limites:**

Les événements ambulance-URG ne constituent pas un marqueur de substitution idéal des besoins en soins aigus dans une population. D’importants déterminants de la santé, notamment les conditions de vie et le statut socioéconomique, étaient absents de l’ensemble de données.

**Conclusion:**

Cette étude met en évidence le fardeau que représente l’utilisation des services ambulanciers pour les receveurs d’une greffe rénale. Elle souligne également la nécessité d’un suivi plus optimal pour certains sous-groupes de patients qui présentent un risque particulièrement élevé.

## Introduction

Kidney transplantation is associated with improved survival and better outcomes compared with dialysis. While transplant recipients experience favorable outcomes, they are still at high risk for increased health care utilization, including emergency department (ED) visits.^1^ A previous study found that among solid-organ transplant recipients (SOTRs), 66.5% of all ED visits occurred in those who received a kidney transplant and the majority of kidney transplant recipients (KTRs) visited the ED within the first 2 years following their transplant.^2^ While there was variability among the types of presentations, the most common presenting complaint for KTRs as well as other SOTRs in the above-mentioned study was abdominal pain and gastrointestinal (GI) symptoms.^3^ Other studies have shown KTRs to be burdened with post-transplant complications including infection; however, they also face burdensome transportation requirements in order to access specialized care.^4,5^

Although previous studies have explored the burden of ED usage for KTRs, to our knowledge, 1 area that has yet to be explored is the utilization of emergency medical services (EMS) for transport to the ED. EMS are providers of first response, out-of-hospital care, and ambulance transport to the ED and provide patient transport between health care centers. The focus on transport to the ED is unique in that it might be representative of more severe clinical presentation and may help to better understand the burden of health care resource utilization preceding hospital presentation. For example, we previously studied ambulance utilization and transport to the ED for dialysis patients and identified that most dialysis patients had at least 1 EMS encounter at some point during their treatment, that the burden of EMS use on the individual was higher with increased age and comorbidities,^6-8^ and that clinical characteristics at the time of ambulance-ED were associated with hyperkalemia in the ED.^9^ To our knowledge, such an analysis has not been conducted in KTRs, which is important as understanding the extent, predictors, and outcomes of EMS use among KTRs may help guide future work to mitigate this risk through novel care pathways or changes to post-transplant follow-up in an effort to reduce health care system burden.

Therefore, the objectives of this study were threefold: (a) to characterize the burden of EMS utilization in a cohort of KTRs and characteristics of patients at presentation, (b) to identify predictors of frequent EMS utilization, and (c) to determine whether patients who experience one or more early ambulance transports to the ED are at higher risk of the composite of death/graft failure, death-censored graft failure (DCGF), and death with graft function (DWGF).

## Methods

### Study Population

We analyzed a retrospective cohort of incident adult (≥18 years), Nova Scotian KTRs affiliated with the Atlantic Canada Multi-Organ Transplant Program (MOTP) who received their first kidney transplant from January 1, 2008 to December 31, 2020, at the Queen Elizabeth II Health Sciences Centre (QEII HSC) in Halifax, Nova Scotia, Canada. Although the MOTP serves of all Atlantic Canada, only those specifically residing in Nova Scotia between the study dates were followed to capture all ambulance-ED events and hospitalizations that are recorded by Emergency Health Services (EHS), the sole provincial provider of EMS in Nova Scotia. Patients were followed until the first of either death or graft loss, loss to follow-up, or until the last date of study (January 1, 2021). Ethics approval was obtained from the Nova Scotia Health Research Ethics Board (file no. 1026828).

### Data Sources, Linkage, and Baseline Characteristics

All data on admission and discharge dates as well as baseline characteristics of KTRs are contained in the Atlantic Canada MOTP database. In our study, KTRs were identified using the MOTP database and linked to the provincial EHS database. Baseline characteristics were captured for all patients. *Recipient characteristics* included age at transplant, sex, body mass index (BMI), recipient Cytomegalovirus (CMV) status, and comorbidities (diabetes mellitus [DM], cerebrovascular disease [CVD], connective tissue disease [CTD], liver disease [LD], congestive heart failure [CHF], coronary artery disease [CAD], peripheral vascular disease [PVD], hypertension, and prior malignancy). Causes of end-stage kidney disease (ESKD) among recipients were also captured and included categories of polycystic kidney disease (PKD), DM, glomerulonephritis, ischemia/hypertension, obstructive/urologic, inherited/non-PKD, other causes, and those of unknown etiology. *Immunological characteristics* included the number of human leukocyte antigen (HLA) mismatches and induction agent (anti-thymocyte globulin, basiliximab, or methylprednisolone alone). *Donor characteristics* included living vs deceased donor (and the latter were further sub-classified as neurological determination of death [NDD] and donation after circulatory death [DCD]), sex, donor age, and donor CMV status. Finally, *perioperative characteristics* included length of stay (LOS), delayed graft function (DGF), warm ischemia time (WIT), and cold ischemia time (CIT). For all patients in this study, baseline characteristics were verified through electronic chart review. As all kidney transplants were performed at a single institution, dates of transplant and discharge were captured using the Canadian Institute for Health Information (CIHI) discharge abstract database (DAD) for the QEII HSC and verified using electronic chart review following a similar process as done previously.^7^

The EHS clinical data collected for this study included day of the week and time of day of transport, chief complaint, first set of vital signs (at the time of paramedic assessment during patient pick-up prior to transport to the ED), and the Canadian Triage and Acuity Scale (CTAS), a scoring system developed to prioritize delivery of patient based on acuity and risk.^10^

### Outcome

The number of ambulance transport events/time at risk was reported as a rate (number/1000 patient days at risk, whereby time at risk was the day of discharge from the index admission for transplantation to cohort exit, as noted above). Graft failure was described as permanent loss of graft function or pre-emptive re-transplant. Mortality was all-cause. All outcomes were captured using the MOTP database as described above; missing outcomes were updated after review of electronic records.

### Analysis

Baseline characteristics were reported for the overall cohort and those who did/did not experience an ambulance transport to ED. Characteristics of each ambulance-ED transport were also reported. Descriptive statistics (number and proportion, mean, standard deviation, median, and interquartile range) were used for all baseline characteristics. The EMS utilization was reported using incidence rate ratios (IRRs; number of events/time at risk) and the cumulative incidence of ambulance-ED (including 95% confidence intervals [CIs]) was visualized using a Kaplan-Meier failure curve.

Predictors of EMS utilization were analyzed using a negative binomial regression model (to account for overdispersion) and included available information in the MOTP database at the time of transplantation, including demographics, comorbidities, transplant procedure characteristics, and immunological factors as noted above. Associations with EMS utilization were reported using IRRs with 95% CIs. Variables used in our negative binomial regression model were chosen based on clinical impression; but in a secondary analysis, we included all available predictors. Time to the composite of death/graft failure, DCGF, and DWGF was analyzed using adjusted Cox survival analyses inclusive of the exposure of early ambulance-ED transport (ie, within 30 days of discharge from hospital after transplantation). Assuming we would only have a limited number of events, in addition to early ambulance transport, only those variables with a significant unadjusted association with graft failure (using a *P* threshold of <.05) were included in this model. We performed pre-specified sensitivity analyses for early ambulance transport at <60, <90, and <180 days from the date of hospital discharge after transplantation and evaluated the same outcomes as noted above.

## Results

**Figure 1.**
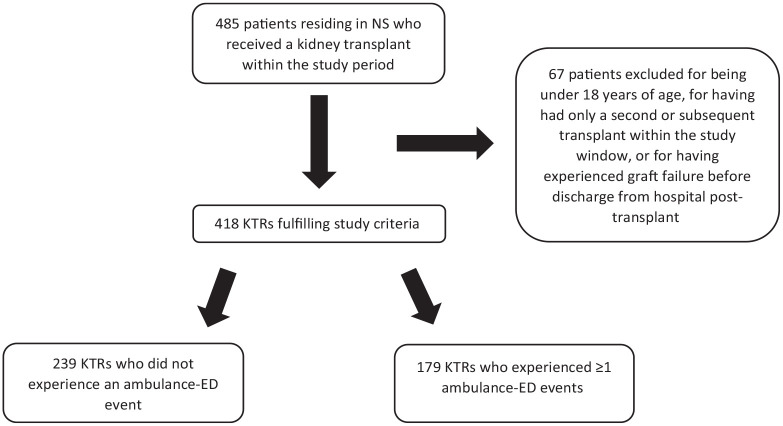
Flow chart outlining exclusions and ambulance-ED events for cohort. *Note.* NS = Nova Scotia; KTR = kidney transplant recipient; ED = emergency department.

Out of a total of 418 KTRs, 179 recipients (42.8%) had 472 ambulance-ED events during the study period (Figure 1). Baseline characteristics of the study population are noted in Table 1. Mean age of the cohort was 52 ± 13 years, 35% were female, and glomerulonephritis was the most common cause of ESKD (30%). To highlight some significant differences between those with and without at least 1 ambulance-ED event; mean age was higher in those with a transport (54.6 vs 49.3 years) and more recipients with a transport were seropositive for CMV at the time of transplant (36% vs 22%). Significant differences were noted across select comorbidities; 33% of those with one or more transports had diabetes at time of transplant compared to 18% of those with no ambulance-ED. Of the 239 patients with no ambulance-ED, 82 (34%) received a kidney from a live donor compared to 44 of the 179 (25%) patients with ≥1 ambulance-ED. Finally, median LOS after transplant was 11 days (interquartile range [IQR] = 8-15) for those with and 9 days (IQR = 8-12) for those without an ambulance-ED.

**Table 1.** Baseline Characteristics of Kidney Transplant Recipients.

	N = 239	N = 179	N = 418	*P*
	No ambulance-ED	Ambulance-ED = ≥1	Total cohort	
Recipient characteristics				
Age at transplant (mean years, SD)	49.1 ± 13.5	54.7 ± 12.5	51.5 ± 13.3	**.000**
Sex (%)				.410
Male	159 (66.53)	112 (62.57)	271 (64.83)	
Female	80 (33.47)	67 (37.43)	147 (35.17)	
Body mass index (kg/m^2^, %)				.689
<18.99	1 (0.43)	1 (0.57)	2 (0.49)	
19-24.99	85 (36.80)	59 (33.91)	144 (35.56)	
25-29.99	76 (32.90)	65 (37.36)	141 (34.81)	
30-34.99	45 (19.48)	27 (15.52)	72 (17.78)	
≥35	24 (10.39)	22 (12.64)	46 (11.36)	
Recipient Cytomegalovirus status (%)	53 (22.18)	65 (36.31)	118 (28.23)	**.002**
Positive	186 (77.82)	114 (63.69)	300 (71.77)	
Negative				
Cytomegalovirus mismatch (%)				.769
Yes	32 (19.63)	116 (82.27)	247 (81.25)	
No	131 (80.37)	25 (17.73)	57 (18.75)	
Comorbidities (%)				
Diabetes	43 (17.99)	60 (33.52)	103 (24.64)	**.000**
Cerebrovascular disease	7 (2.93)	10 (5.59)	17 (4.07)	.213
Connective tissue disease	8 (3.54)	5 (2.91)	13 (3.27)	.784
Liver disease	16 (7.08)	14 (8.14)	30 (7.54)	.705
Congestive heart failure	11 (4.60)	5 (2.79)	16 (3.83)	.443
Coronary artery disease	14 (5.86)	17 (9.50)	31 (7.42)	.188
Peripheral vascular disease	12 (5.02)	13 (7.26)	25 (5.98)	.406
Hypertension	236 (100.00)	177 (98.88)	413 (99.52)	.185
Prior Malignancy	23 (9.62)	22 (12.29)	45 (10.77)	.427
End-stage kidney disease cause (%)				**.001**
Polycystic kidney disease (PKD)	44 (18.41)	35 (19.55)	79 (18.90)	
Diabetes	28 (11.72)	49 (27.37)	77 (18.42)	
Glomerulonephritis	82 (34.31)	42 (23.46)	124 (29.67)	
Ischemia/hypertension	18 (7.53)	14 (7.82)	32 (7.66)	
Obstructive/urologic	14 (5.86)	16 (8.94)	30 (7.18)	
Non-PKD inherited	22 (9.21)	6 (3.35)	28 (6.70)	
Other	15 (6.28)	10 (5.59)	25 (5.98)	
Unknown	16 (6.69)	7 (3.91)	23 (5.50)	
Immunological Characteristics				
Number of HLA mismatches (%)				.404
0	12 (5.50)	6 (3.51)	18 (4.63)	
1	5 (2.29)	9 (5.26)	14 (3.60)	
2	32 (14.68)	16 (9.36)	48 (12.34)	
3	48 (22.02)	35 (20.47)	83 (21.34)	
4	43 (19.72)	40 (23.39)	83 (21.34)	
5	44 (20.18)	38 (22.22)	82 (21.08)	
6	34 (15.60)	27 (15.79)	61 (15.68)	
Induction type (%)				**.014**
Anti-thymocyte globulin	44 (20.66)	54 (33.13)	98 (26.06)	
Basiliximab	162 (76.06)	107 (65.64)	269 (71.54)	
Methylprednisolone	7 (3.29)	2 (1.23)	9 (2.39)	
Donor characteristics				
Donor type (%)				
Living	82 (34.31)	44 (24.58)	126 (30.14)	**.040**
Deceased	157 (65.69)	135 (75.42)	292 (69.86)	.429
Neurologic determination of death	134 (85.35)	110 (81.48)	244 (83.56)	
Donation after circulatory death	23 (14.65)	25 (18.52)	48 (16.44)	
Sex (%)				.489
Male	111 (46.64)	90 (50.28)	201 (48.20)	
Female	127 (53.36)	89 (49.72)	216 (51.80)	
Age of donor (mean years, SD)	44.1 ± 15.9	48.3 ± 14.5	45.9 ± 15.4	**.006**
Donor Cytomegalovirus status (%)				.307
Positive	41 (25.15)	43 (30.50)	84 (27.63)	
Negative	122 (74.85)	98 (69.50)	220 (72.37)	
Perioperative characteristics				
Length of stay (median days, IQR)	9 (8-12)	11 (8-15)	10 (8-13)	**.000**
Delayed graft function (%)				.08
Yes	30 (14.2)	35 (21.3)	65 (17.3)	
No	182 (85.8)	129 (78.7)	311 (82.7)	
Warm ischemia time (median minutes, IQR)	30 (23-40.5)	30 (24-44)	30 (24-42)	.256
Cold ischemia time (median minutes, IQR)	390 (185-630)	412.5 (225-573.5)	405 (210-610)	.832

*Note.* ED = emergency department; HLA = human leukocyte antigen; IQR = interquartile range.Bold values represent *P* < 0.05 significance.

**Figure 2.**
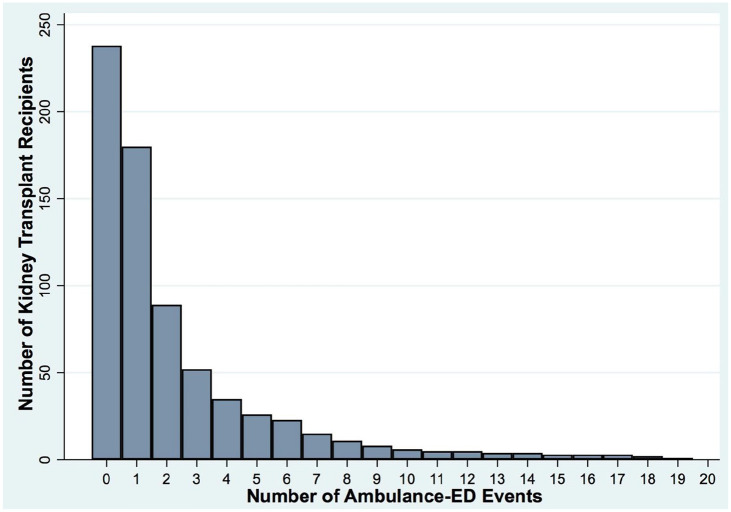
Number of ambulance-ED events among kidney transplant recipients. *Note.* ED = emergency department.

**Figure 3.**
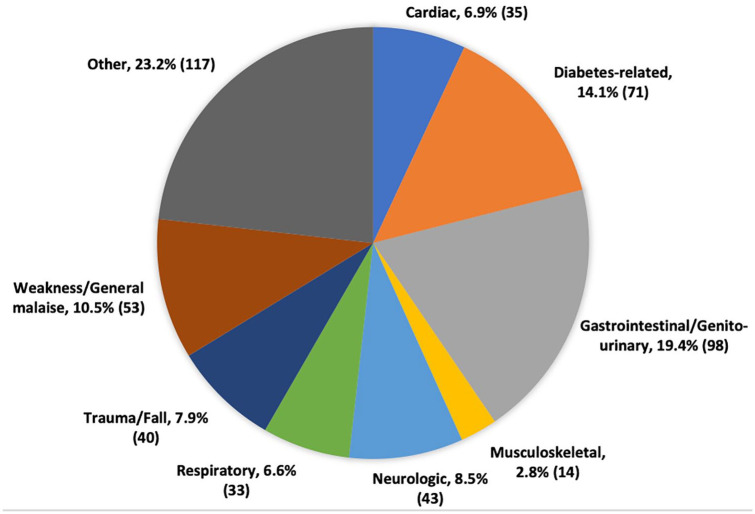
Presenting complaint at time of ambulance-ED. *Note.* ED = emergency department.

Characteristics of patients for each ambulance-ED are described in Table 2, and the number of ambulance-ED events among all KTRs is visually depicted in Figure 2. There were anywhere from 1 to 19 ambulance-ED events for the 179 patients with one or more transports. Mean age and CTAS score at time of ambulance-ED was 57.9 ± 12.8 years and 2.9 ± 1.0, respectively. Out of all ambulance-ED with a recorded CTAS (N = 443), just over 3 quarters (75.4%) were either CTAS 2 (emergent care within 15 minutes) or CTAS 3 (urgent care within 30 minutes). A total of 12 (2.7%) transports were deemed to be CTAS 1 (requiring immediate resuscitation), and the rest were categorized as either less urgent or non-urgent (21.9%).^10^ Mean and median time to first ambulance-ED for the cohort was 3.2 ± 2.9 years and 2.4 (0.9-4.6) years, respectively. Figure 3 depicts the presenting complaint at the time of ambulance-ED. The most common presenting complaint by body system was GI/Genitourinary (18.4%). Of the total ambulance-ED events, 339 (71.8%) took place during the week compared to 133 (28.2%) that occurred over the weekend. There was a near equal distribution with respect to time of day for ambulance-ED transport, with 233 (49.4%) occurring during the daytime and 239 (50.6%) occurring during after-hours (between 17:00 and 08:00).

**Table 2.** Characteristics of Kidney Transplant Recipients at Time of Ambulance-ED.

Characteristic	Value
Total number of ambulance-ED events for cohort (range)	472 (1-19)
Number of patients with ≥1 ambulance-ED (%)	179 (42.8)
Age at ambulance-ED (mean, SD)	57.9 ± 12.8
Sex (%)	
Male	112 (62.6)
Female	67 (37.4)
Canadian triage and acuity scale (mean, SD)	
1 (%)	2.9 ± 1.0
2 (%)	12 (2.7)
3 (%)	148 (33.4)
4 (%)	186 (42.0)
5 (%)	58 (13.1)
Vital signs	39 (8.8)
Temperature (mean °C, SD)	36.8 ± 1.1
≥40 (%)	<5 (<1)
≤35 (%)	10 (3.1)
Systolic blood pressure (mean mm Hg, SD)	133.8 ± 33.6
≥180 (%)	39 (7.9)
≤80 (%)	29 (5.9)
Diastolic blood pressure (mean mm Hg, SD)	74.6 ± 18.3
Heart rate (mean beats/min, SD)	88.3 ± 23.7
≥130 (%)	20 (4.0)
≤40 (%)	10 (2.0)
Respiratory rate (mean breaths/min, SD)	19.3 ± 5.4
≥30 (%)	28 (5.6)
≤8 (%)	9 (1.8)
Oxygen saturation (median, IQR)	98 (96-99)
≤85 (%)	18 (3.8)
Time to first ambulance-ED from discharge following transplant	
(Mean years, SD)	3.2 ± 2.9
(Median years, IQR)	2.4 (0.9-4.6)
Day of week transported	
Weekday	339 (71.8)
Weekend	133 (28.2)
Time of day transported	
Daytime	233 (49.4)
After-hours	239 (50.6)

*Note.* ED = emergency department; IQR = interquartile range.

The cumulative incidence of ambulance-ED events among KTRs is depicted in Supplemental Figure 1. Ambulance-ED transports tended to occur with increasing incidence late following transplant, particularly at the 7-year mark and beyond. Incident rate ratios for ambulance-ED events based on baseline characteristics are shown in Table 3. For recipient characteristics, female sex (IRR = 1.60; 95% CI = 1.12-2.29) and end-stage kidney disease due to diabetes (IRR = 2.52; 95% CI = 1.19-5.31) were associated with increased incidence of ambulance-ED. For donor characteristics, receiving a kidney from a donor age ≥45 years was also associated with increased incidence of ambulance-ED (IRR = 1.50; 95% CI = 1.04-2.15). There were no other statistically significant associations with ambulance-ED.

**Table 3.** Predictors of Ambulance Transport to the Emergency Department (N = 643 Events).

	Incidence rate ratio	95% confidence interval	*P*
Recipient characteristics			
Female sex	**1.60**	**1.12 to 2.29**	**.010**
Age at transplant (≥65 versus <65 years)	1.52	0.96 to 2.42	.077
Diabetes	1.64	0.83 to 3.27	.157
Malignancy	0.76	0.41 to 1.38	.364
Coronary artery disease	0.85	0.44 to 1.63	.620
Cause of end-stage kidney disease related to diabetes	**2.52**	**1.19 to 5.31**	**.015**
Immunological characteristics			
Induction type			
Anti-thymocyte globulin	Ref	–	−
Basiliximab	1.03	0.69 to 1.55	.888
Methylprednisolone	1.46	0.44 to 4.82	.531
Donor characteristics			
Live donor	0.87	0.58 to 1.32	.512
Female sex	1.07	0.76 to 1.51	.706
Age ≥45 years	**1.50**	**1.04 to 2.15**	**.030**
Perioperative characteristics			
Length of stay ≥ 10 days	1.45	0.99 to 2.13	.060
Delayed graft function	1.50	0.94 to 2.40	.092
Warm ischemia time (minutes)	1.00	0.99 to 1.02	.708

Bold values represent *P* < 0.05 significance.

Patients with one or more ambulance-ED events were observed to have a higher proportion of the composite of death/graft failure compared to those with no ambulance-ED; of the 179 patients who had one or more ambulance-ED events, 117 (65.4%) experienced death/graft failure vs 37 (15.5%) of those who did not experience an ED transport. After multivariable adjustment, there was no significant increase in the composite of death/graft failure, DWGF, or DCGF among those experiencing an ambulance-ED event within 30 days of discharge from transplantation (N = 14). This lack of association was observed in all additional sensitivity analyses (Table 4) with the exception of DWGF following ambulance-ED within 180 days (hazard ratio [HR] = 2.16; 95% CI = 1.10-4.26). Other factors associated with the composite of death/graft failure included female donor sex (HR = 1.57; 95% CI = 1.00-2.47), donor age ≥45 (HR = 2.17; 95% CI = 1.30-3.61) and donor type (live vs deceased, HR = 0.57, 95% CI = 0.33-0.99, Supplemental Table 1).

**Table 4.** Risk of Graft Failure or Death for Those That Experienced One or More Ambulance-ED Events versus Those Without an Ambulance-ED (N = 399).

Model	Hazard ratio	95% confidence interval	*P*
Adjusted model^a^			
Graft failure or death (N = 97)			
Ambulance-ED within			
• 30 days of discharge (N = 14)	1.20	0.44-3.27	.721
• 90 days of discharge (N = 28)	1.24	0.57-2.67	.589
• 180 days of discharge (N = 142)	1.42	0.76-2.66	.275
Death-censored graft failure (N = 42)			
Ambulance-ED within			
• 30 days of discharge	1.36	0.33-5.64	.671
• 90 days of discharge	0.78	0.19-3.24	.734
• 180 days of discharge	0.55	0.13-2.27	.406
Death with graft function (N = 62)			
Ambulance-ED within			
• 30 days of discharge	1.44	0.45-4.60	.538
• 90 days of discharge	1.72	0.74-4.01	.205
• 180 days of discharge	2.16	1.10-4.26	**.026**

*Note.* ED = emergency department.

a Adjusted for recipient sex, age at transplant (≥65 vs <65 years), diabetes, malignancy, coronary artery disease, end-stage kidney disease secondary to diabetes, induction type, live donor status, donor sex, donor age ≥45 years, length of stay ≥10 days, delayed graft function, and warm ischemia time.Bold values represent *P* < 0.05 significance.

## Discussion

In this study, we identified that KTRs experienced a high rate of ambulance transport to the ED (particularly late after their initial transplant), most notably for reasons related to a GI or genitourinary cause. We found several baseline characteristics that were associated with greater ambulance-ED including female sex, ESKD secondary to diabetes, and older donor age. Finally, while there was a high burden of ambulance use, early ambulance-ED was not associated with graft loss or mortality among KTRs in this study.

Although this is the first study (to our knowledge) exploring predictors and outcomes of ambulance-ED among KTRs, our results generally align with prior studies exploring the burden of ED use among KTRs. Our study findings were similar to those in the literature showing increased ED use among recipients with diabetes and of female sex.^2,11^ In our adjusted models, female sex was associated with a higher risk of ambulance-ED among recipients. There is conflicting evidence on the role of sex with respect to kidney allograft outcomes. Many but not all studies have found differences in graft survival between males and females based on donor sex, donor and patient age, as well as socioeconomic status^12-19^ often explained by differences in immunological response between the sexes among other factors.^20-22^ One other potential reason that could be contributing to the difference in ambulance-ED observed between females and males in our study is the difference in threshold of EMS activation when comparing male vs female sex. In 1 study looking at EMS activation within a cohort of patients with acute coronary syndromes, females were more likely to activate EMS than males,^23^ and in a US study looking at statewide EMS use, it was found that women were statistically more likely to present to EMS with illnesses compared to males.^24^ These observed differences may explain why even though the majority of our recipient cohort was male, ambulance-ED was relatively higher among females.

In addition to sex, we found that ESKD secondary to diabetes was strongly associated with ambulance-ED vs those without. This is consistent with the established association between diabetes and poor health outcomes in the post-transplant period seen in the literature.^25^ In contrast, we found that prolonged hospital LOS and DGF were not associated with increased ambulance-ED in our study, although the lower bound of the CI (particularly for LOS) may suggest that this was related to our sample size. Nonetheless, this is somewhat inconsistent with the literature regarding the impact of these factors on other outcomes for KTRs.^11^ Beyond sample size, 1 possible explanation is that events that would normally require ED transport may occur during complicated and prolonged hospital admissions (some of which may occur concurrently with DGF). These events would not be included in our analysis, which focused only on events that occurred after the initial transplant hospitalization.

While our study is consistent with the findings of studies evaluating ED presentation (irrespective of mode of transport), younger age was not associated with a significantly higher risk of ambulance-ED, which is in contrast to the literature suggesting a higher incidence of ED visits among younger KTRs.^2^ We surmise that ambulance transport to the ED likely reflects the transport of a patient who is unable to access the ED by their own means, suggesting a more serious presentation. Older adults, due in part to other factors such as comorbidity, mobility, and frailty, may be more likely to require ambulance-ED even for less severe presentations. This is somewhat reflected in the point estimate of 1.52 for age ≥65 years and ambulance-ED (which suggests a higher risk for older adults), although this was not statistically significantly different in our study.

In this study, we observed that recipients who received a kidney from a donor ≥45 years of age were associated with increased ambulance-ED. This is in keeping with existing literature showing that increased donor age is associated with reduced long-term allograft survival.^26^ Interestingly, receiving a kidney from a live versus deceased donor was not associated with a lower risk for ambulance-ED. This was a surprising finding given the well-established patient and graft survival benefit with live vs deceased donor organs.^27-30^ We hypothesize that this may relate in part to the approach to care for transplant recipients early after transplantation at our center. Many patients (both live and deceased) are frequently reassessed as outpatients through the transplant clinic subsequent to transplant, and those with changes in health status may be directly admitted without need for ambulance-ED. This approach may be more common for deceased donor recipients who are in turn, more likely to experience post-operative complications identified through a clinic requiring direct admission.^31^

This study allowed us to identify characteristics that were associated with increased acute care needs among the KTR population and highlighted the burden of care on KTRs after their transplant. The findings from our study emphasize the importance of recognizing existing risk factors in KTRs toward a goal of providing individualized, patient-centered care. For example, among higher-risk KTRs, prolonged close follow-up post-transplant and increased use of virtual care visits could be implemented to monitor these patients to pre-emptively avoid ambulance-ED. Interestingly, many first ambulance-ED transports occurred well after the initial transplantation. Once again, this may reflect our institutional practice of direct admission, particularly for patients who are fresh post-transplantation (as noted above) and suggests that this could be a strategy to mitigate the burden of ambulance-ED transports. Alternatively, this may reflect the well-documented period of “high-risk” among individuals with a failing graft.^32^ This emphasizes the need for continued close monitoring of patients even far-removed from their initial transplant due to their risk of adverse outcomes.

This study does have some important limitations. We used multiple variables in our adjusted models based on clinical importance and availability; however, we were limited in our approach to variable inclusion, particularly for survival analyses (due to the limited number of outcomes). Another limitation of this study was that ambulance-ED is not a perfect surrogate marker for acute care needs of a population. Distress and care needs that cause an individual to call for an ambulance can vary from person to person based on one’s perception of their own illness acuity and access to other ways to be admitted to hospital (ie, direct through transplant clinic). Living situation, eg, living alone vs living with supports, could play an important role in whether one activates EMS and was not explored in this study. It is important to note that self-transportation or direct admission from the transplant clinic would not fall under ambulance-ED, so it is possible that the burden of ambulance-ED would be higher in centers where direct admission is not a common occurrence. It is also well-known that socioeconomic status as well as geographic location (including rurality) and easy access to health care centers is an important determinant of health, and this, alongside medical literacy, was not explored for our cohort of due to lack of availability of data. A further point to note is that while we had a representative population of transplant recipients from a large geographic region, our sample size was relatively small, and thus, CIs were wide. It is possible that with a larger sample, other factors may have been associated with ambulance-ED (including older recipient age, prolonged length of hospital stay, and DGF). Finally, while we did have information on some donor factors, we lacked the data granularity to delineate between expanded and standard criteria donors (ECDs) and did not have access to information to determine kidney donor profile index (KDPI) scores, known predictors of poor outcomes in kidney transplantation.

## Conclusions

In this study, we found the need for ambulance transport to the ED was common in the post-kidney transplant population, and that several factors were associated with a higher risk of ambulance-ED. These predictive factors may give insight into the need for more optimal follow-up, especially in those patient subgroups who are most affected.

## Supplemental Material

sj-docx-1-cjk-10.1177_20543581251324587 – Supplemental material for Ambulance Service Utilization by Kidney Transplant Recipients Supplemental material, sj-docx-1-cjk-10.1177_20543581251324587 for Ambulance Service Utilization by Kidney Transplant Recipients by Kaveh Masoumi-Ravandi, Amanda Vinson, Aran Thanamayooran, Judah Goldstein, Thomas Skinner and Karthik Tennankore in Canadian Journal of Kidney Health and Disease
