# Supplementary material for: Ambulance Service Utilization by Kidney Transplant Recipients
Source: Can J Kidney Health Dis. 2025 Apr 4;12:20543581251324587. doi: 10.1177/20543581251324587 (PMC11970094; doi:10.1177/20543581251324587)
Supplement: sj-docx-1-cjk-10.1177_20543581251324587 – Supplemental material for Ambulance Service Utilization by Kidney Transplant Recipients [file sj-docx-1-cjk-10.1177_20543581251324587.docx]

**Supplemental Table 1. Factors associated with death/graft failure for those experiencing an ambulance-ED within 30 days (N=418 individuals, N=99 events)**

|  | **Hazard Ratio** | **95% Confidence Interval** | ***P* value** |
| --- | --- | --- | --- |
| **Recipient Characteristics** |  |  |  |
| Sex (female vs male) | 1.08 | 0.69-1.70 | 0.731 |
| Age at transplant (≥65 versus <65 years) | 1.01 | 0.58-1.76 | 0.965 |
| Diabetes | 1.61 | 0.76-3.39 | 0.213 |
| Malignancy | 1.30 | 0.69-2.46 | 0.413 |
| Coronary artery disease | 1.49 | 0.71-3.14 | 0.296 |
| Cause of end-stage kidney disease related to diabetes | 1.16 | 0.50-2.69 | 0.737 |
| **Immunological Characteristics** |  |  |  |
| Induction type  Anti-thymocyte globulin Basiliximab Methylprednisolone | Ref 0.78  0.86 | -- 0.48–1.27  0.19–3.81 | -- 0.312  0.838 |
| **Donor Characteristics** |  |  |  |
| Live donor | **0.57** | **0.33-0.99** | **0.048** |
| Female sex | **1.57** | **1.00–2.47** | **0.049** |
| Age ≥ 45 years | **2.17** | **1.30–3.61** | **0.003** |
| **Perioperative Characteristics** |  |  |  |
| Length of stay ≥ 10 days | 1.44 | 0.88-2.35 | 0.148 |
| Delayed graft function | 1.57 | 0.90-2.73 | 0.114 |
| Warm Ischemia Time (minutes) | 1.00 | 0.99–1.01 | 0.885 |
| Ambulance-ED within 30 days of discharge | 1.31 | 0.44-3.94 | 0.627 |

**
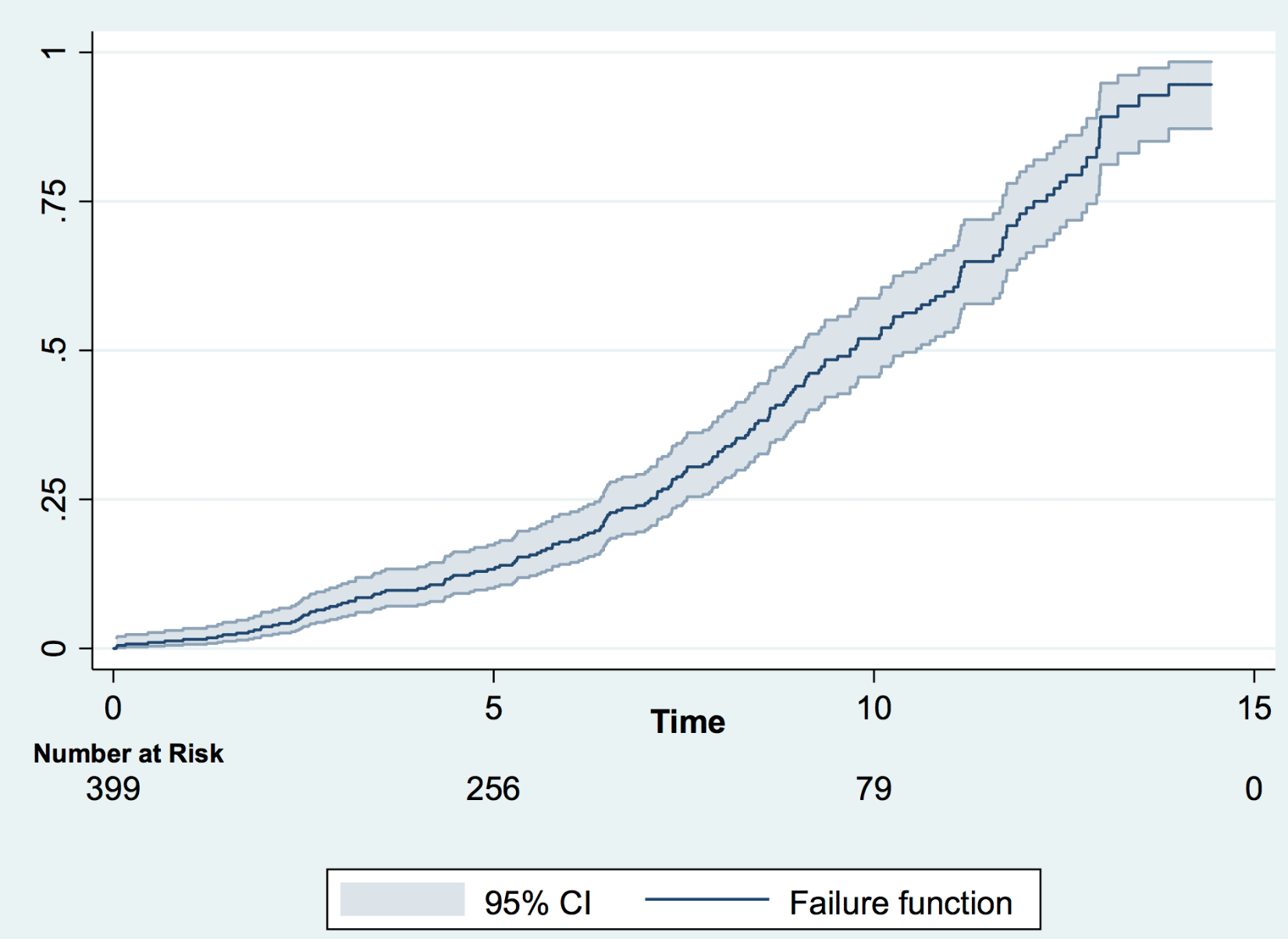
**

**Supplemental Figure 1. Cumulative incidence of ambulance-ED with a Kaplan-Meier failure curve**
